# A new species of *Neoantistea* Gertsch, 1934 from Yintiaoling National Nature Reserve, Chongqing, China (Araneae, Hahniidae)

**DOI:** 10.3897/BDJ.13.e155306

**Published:** 2025-05-09

**Authors:** Changbin Zheng, Yannan Mu, Luyu Wang

**Affiliations:** 1 Management Center of Yintiaoling National Nature Reserve, Chongqing City, China Management Center of Yintiaoling National Nature Reserve Chongqing City China; 2 Southwest University, Chongqing City, BeiBei District, China Southwest University Chongqing City, BeiBei District China

**Keywords:** classification, description, diversity, morphology, taxonomy

## Abstract

**Background:**

Hahniidae is a family of spiders with 239 species belonging to 29 genera, of which 45 species belong to 9 genera was recorded in China.

**New information:**

A new species of the genus *Neoantistea* Gertsch, 1934 is described from Yintiaoling Nature Reserve: *N.yintiaoling* **sp. nov.** (♂). Morphological descriptions, photos and illustrations of copulatory organs are provided. Photos of *N.quelpartensis* Paik, 1958 are also presented in order to compare it with new species.

## Introduction

The spider family Hahniidae is distinguished from other spider families by its comb-like spinnerets. It represents one of the small spider groups, comprising 29 genera and 239 species worldwide, with 9 genera and 45 species recorded in China (WSC 2025). There is only one species of *Neoantistea* recorded from China, *N.quelpartensis* Paik, 1958 (Figs 4, 5).

In this paper, a new species of the genus *Neoantistea* from Yintiaoling Nature Reserve, northeast Chongqing are described: *N.yintiaoling* sp. nov. (♂). All speciemens of this new species are collected in leaf litter.

## Materials and methods

All specimens are preserved in 75% ethanol and were examined, illustrated, photographed and measured using a Leica M205A stereomicroscope equipped with a drawing tube, a Leica DFC450 Camera and LAS software (Ver. 4.6). Male pedipalps and epigynes were examined and illustrated after they were dissected. Eye sizes were measured as the maximum dorsal diameter. Leg measurements are shown as: total length (femur, patella and tibia, metatarsus, tarsus). All measurements are in millimetres. Specimens examined here are deposited in the Collection of Spiders, School of Life Sciences, Southwest University, Chongqing, China (SWUC).

Abbreviations used in the text: **ALE**–anterior lateral eye; **AME**–anterior median eye; **PLE**–posterior lateral eye; **PME**–posterior median eye.

## Taxon treatments

### Neoantiste yintiaoling sp. nov.

A31C7239-35BA-5137-8794-D5E5DDFC43F4

08DEFBA1-6794-40F0-B4A6-5BA34A8500EC

#### Materials

- **Type status:** Holotype. **Occurrence:** individualCount: 1; sex: male; lifeStage: adult; occurrenceID: 95795905-6A1F-5242-AA34-7A464CD2ADD0; **Taxon:** scientificName: *Neoantisteayintiaoling*; order: Araneae; family: Hahniidae; genus: Neoantistea; **Location:** country: China; stateProvince: Chongqing; county: Wuxi; locality: Yintiaoling Nature Reserve, Luomadian, Huanglianchanggou; verbatimElevation: 1467; verbatimLatitude: 31°32′29″N; verbatimLongitude: 109°51′00″E; **Event:** year: 2024; month: 8; day: 11; **Record Level:** institutionID: the Collection of Spiders, Southwest University; institutionCode: SWUC
- **Type status:** Paratype. **Occurrence:** individualCount: 3; sex: male; lifeStage: adult; occurrenceID: 815BB523-71C7-5358-9C36-FF6C1D5662CD; **Taxon:** scientificName: *Neoantisteayintiaoling*; order: Araneae; family: Hahniidae; genus: Neoantistea; **Location:** country: China; stateProvince: Chongqing; county: Wuxi; locality: Yintiaoling Nature Reserve, Luomadian, Huanglianchanggou; verbatimElevation: 1467; verbatimLatitude: 31°32′29″N; verbatimLongitude: 109°51′00″E; **Event:** year: 2024; month: 8; day: 11; **Record Level:** institutionID: the Collection of Spiders, Southwest University; institutionCode: SWUC

#### Description

**Figure 1.**
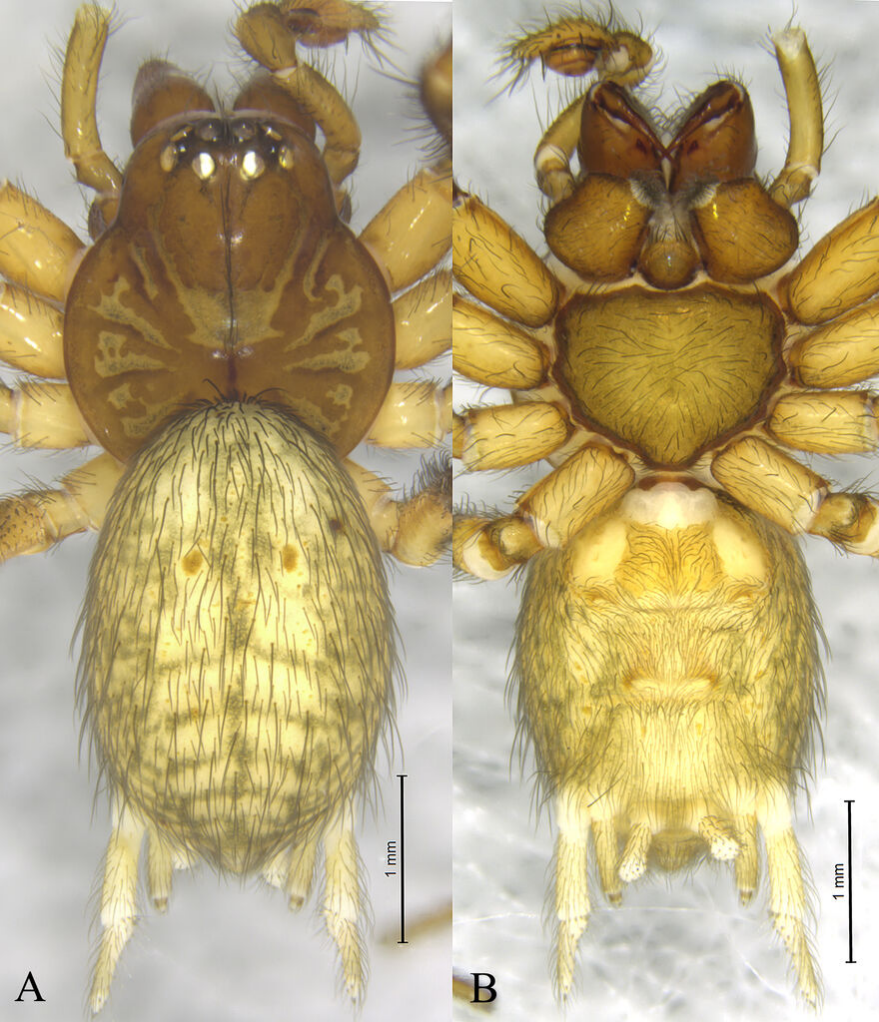
*Neoantisteayintiaoling* sp. nov., male holotype. **A** Male habitus, dorsal view; **B** Same, ventral view.

Males total length 3.25–4.78. Male holotype (Fig. 1) total length 4.54. Prosoma 2.22 long, 1.98 wide; Opisthosoma 2.80 long, 1.84 wide. Carapace brown, irregular-shaped, with yellowish-brown radial markings extending from the fovea. Fovea vertical. Cervical groove and radial furrows distinct. Eye sizes and interdistances: AME 0.14, ALE 0.18, PME 0.16, PLE 0.17; AME–AME 0.06, AME–ALE 0.04, PME–PME 0.15, PME–PLE 0.08, ALE–PLE 0.03. MOA 0.40 long, anterior width 0.35, posterior width 0.45. Clypeus height 0.24. Chelicerae, brown, with three promarginal and two retromarginal teeth. Labium brown, wider than long. Endites brown, longer than wide. Sternum yellowish-brown and scutellate with sparse black hairs. Leg measurements: I 10.06 (2.89, 3.48, 2.31, 1.38); II 7.92 (2.23, 2.70, 1.80, 1.19); III 6.66 (1.85, 2.20, 1.52, 1.09); IV 7.93 (2.03, 2.57, 1.99, 1.34). Leg formula: 1423. Opisthosoma oval, dorsum yellowish-brown, dorsally with five light chevrons, venter yellowish-brown.

**Figure 2.**
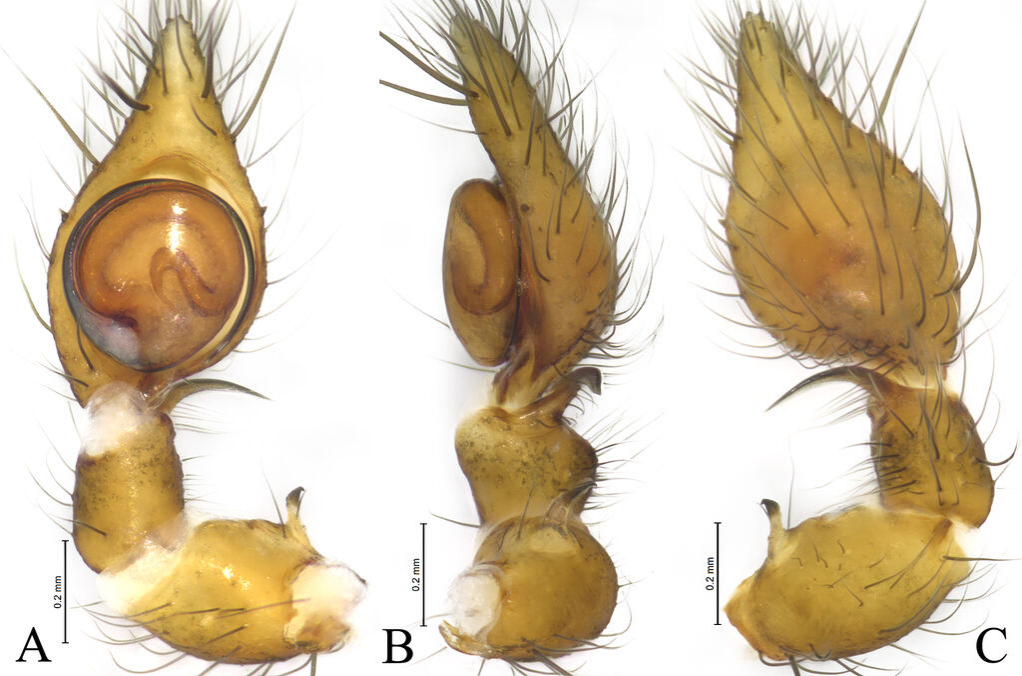
*Neoantisteayintiaoling* sp. nov., male holotype. **A** Left male palp, ventral view; **B** Same, retrolateral view; **C** Same, dorsal view.

**Figure 3.**
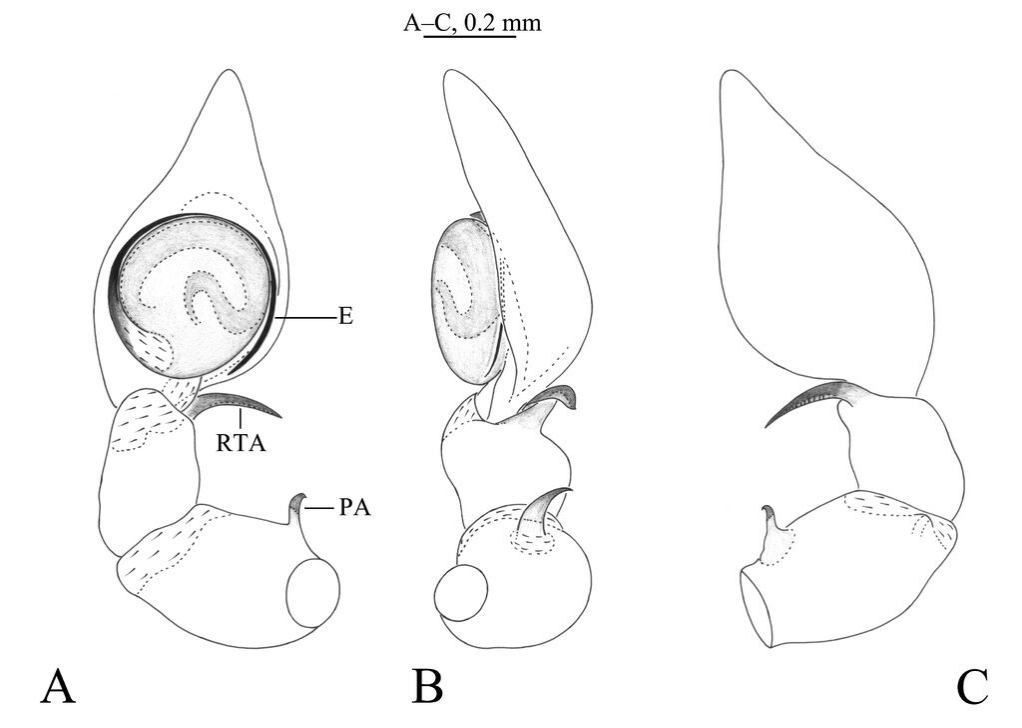
*Neoantisteayintiaoling* sp. nov., male holotype. **A** Left male palp, ventral view; **B** Same, retrolateral view; **C** Same, dorsal view. Abbreviations: E—embolus; PA—patellar apophysis; RTA—retrolateral tibial apophysis.

Palp (Figs 2, 3). Patella with short apophysis (PA), curving and with a hook end. Retrolateral tibia apophysis (RTA) arc-shaped in ventral and dorsal view, helical-shaped in retrolateral view. The embolus (E) originating at about 6:00 o’clock position, clockwise curved along the bulbus margin.

Female. Unknown.

#### Diagnosis

**Figure 4.**
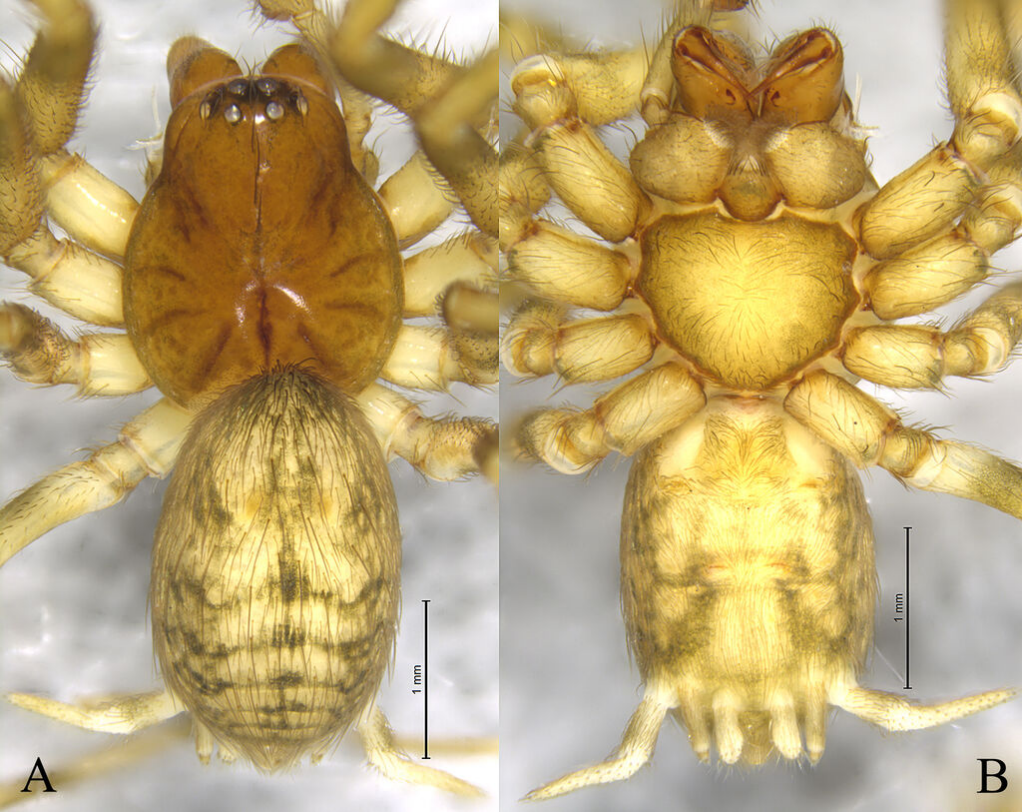
*Neoantisteaquelpartensis* Paik, 1958 (from Hunchun City, Jilin Province, China), male. **A** Male habitus, dorsal view; **B** Same, ventral view.

**Figure 5.**
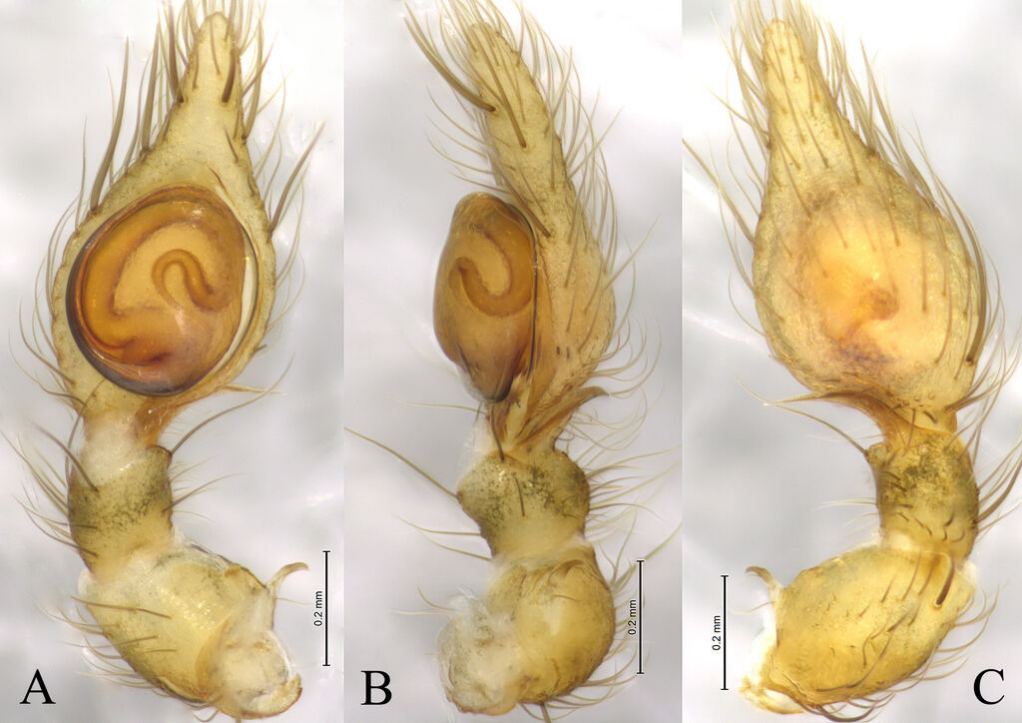
*Neoantisteaquelpartensis* Paik, 1958 (from Hunchun City, Jilin Province, China). **A** Left male palp, ventral view; **B** Same, retrolateral view; **C** Same, dorsal view.

The new species is similar to *N.quelpartensis* Paik, 1958 (Figs 4, 5) in having similarly shaped patellar and tibia retrolateral apophyses, but it can be distinguished by the rounded tegulum (vs. oval, cf. Fig. 2A, Fig. 3A and Fig. 5A), the embolus originating at about 6:00 o’clock position (vs. 5:30, cf. Fig. 2A, Fig. 3A and Fig. 5A), the strong patellar apophysis (vs. puny, cf. Figs 2, 3 and Fig. 5).

#### Etymology

The specific name is derived from the type locality; noun in apposition.

#### Distribution

Known only from the type locality.

#### Biology

Living under stones by the stream, weaves a small net.

## Supplementary Material

XML Treatment for Neoantiste yintiaoling
